# Patient delay in seeking tuberculosis diagnosis and associated factors in Hadiya Zone, Southern Ethiopia

**DOI:** 10.1186/s13104-018-3215-y

**Published:** 2018-02-09

**Authors:** Terefe Gone Fuge, Solomon Gebre Bawore, Deneke Wolde Solomon, Tadele Yohannes Hegana

**Affiliations:** 1 Department of Medical Laboratory Sciences, Hosanna College of Health Sciences, P.O. BOX 159, Hosanna, Ethiopia; 2 Department of Public Health, Hosanna College of Health Sciences, Hosanna, Ethiopia

**Keywords:** Tuberculosis diagnosis, Patient delay, Associated factors

## Abstract

**Objective:**

To assess patient delay in seeking tuberculosis diagnosis and associated factors in Hadiya Zone, Southern Ethiopia.

**Results:**

The median patient delay in tuberculosis diagnosis in Hadiya Zone was found to be 30 days. Socioeconomic and perception related factors were identified as independent predictors for tuberculosis diagnosis delay. Socioeconomic characteristics like urban residence [OR 2.36; CI 1.64–3.40], religious views [OR 1.24; CI 1.73–7.0], low monthly income [OR 3.38; CI 2.01–5.66] were statistically significantly associated with patient delay in tuberculosis diagnosis. On the other hand, attitudinal determinants such as misconception about the time of TB treatment to be cured and lack of comfort with directly observed treatment short course service [OR 1.54; CI 1.02–2.30] were identified as independent predictors of patient delay in tuberculosis diagnosis. Thus, there is a need for more robust information dissemination strategy to ultimately change people’s views that tuberculosis can only be cured when diagnosed and treated promptly.

## Introduction

Despite the implementation of decentralized tuberculosis control programs such as directly observed treatment short course (DOTS), TB remains to be a major public health threat particularly in resource limited countries including Ethiopia [1]. Tuberculosis early case detection and prompt treatment of infectious cases are believed to be the basis to reverse the incidence of the disease. This can be promulgated by the fact that most of the tuberculosis transmissions occur between onset of TB symptoms and initiation of treatment [2].

Nevertheless, TB case detection rate in Ethiopia was 67.3% in 2015, which was more than 2014 achievement (53.7%) but below the target set by ministry of health and WHO. The detection rate also highly varies from region to region as well as from district to district in the country where some regions are fairly achieving the goal while others remain sluggish [3]. Furthermore, the country’s tuberculosis control programs depends on passive case detection which is based on diagnosing infectious cases whom present themselves to the health facilities which is highly dependent on patient motivation and knowledge, financial capability, degree of suspiciousness of health workers, and the accuracy and effectiveness of diagnostic services [4].

It has been reported that in almost all developing countries, the national TB control programs are able to detect on average half of smear-positive tuberculosis cases using this method. The remaining half will thus continue to transmit TB infection in the community until detected by other health sectors. In this regard, a single case of untreated smear positive tuberculosis can infect up to 15 people annually and over 20 during the natural course of untreated disease [5]. Therefore, to strengthen the passive case detection strategy which is most cost effective and also most appropriate for developing countries like Ethiopia, the bottlenecks that hinder its effectiveness must be understood well.

## Main text

### Methods

#### Study area

This was a cross sectional facility based study conducted in Hadiya Zone, Southern Ethiopia between May and September, 2016. Hadiya Zone is located 232 km South West of Addis Ababa, the capital of Ethiopia. For administrative purposes, the zone is structured into 10 districts and a Town Administration. All the districts are providing tuberculosis diagnosis and treatment service including DOTS at the nearest possible health facilities to the patient which is mainly in health centers and health posts.

#### Sample size estimation and sampling technique

By using a formula for the estimation of a proportion: n = Z^2^. P(1 − P)/e^2^ [9] and P = 0.62, proportion of delay taken from previous study in Northern Ethiopia [6], 95% CI (1.96), 5% margin of error (e) and contingency for non-response rate of 10%; a maximum of 398 study participants were required. To obtain this, four districts were randomly selected out of ten districts found in the zone besides a general hospital which provides a referral service. Then, a sample of two health centers were randomly taken from all health facilities found in each district.

All TB patients who were diagnosed within 3 months prior to the day of interview and able to provide the information were enrolled and interviewed using a structured questionnaire which included close-ended questions. To ensure the enrollment of all TB patients, patients who had been diagnosed 3 months prior to the day of interview were identified using registers before commencing data collection activities. TB patients who were just being diagnosed when the interview was going on were also enrolled. Date of onset of pulmonary symptoms and date of first visit to a health facility was asked. If a patient didn’t remember the exact dates, he/she was asked if it was at the beginning of the month, at mid-month or at end of the month.

#### Data analysis

Data were computerized using Epidata version 3.1 data entry format and exported to statistical software, STATA version 11 for analysis. Independent variables such as socio-demographic characteristics, behavioral and awareness related factors were initially tested for association with patient TB diagnosis delay by using the binary logistic regression model. Those variables which showed a statistical association of P < 0.2 were put in the multivariable analysis model to check if the association existed after controlling possible confounders. Then, all statistical tests and generalizations were done by assuming 95% confidence interval and 5% level of significance (P < 0.05).

### Results

#### Socioeconomic characteristics

Out of 398 tuberculosis patients involved in the study, 395 (99.3%) of them provided complete information. 224 (56.7%) of the patients were males and 171 (43.3%) of them were females. The mean age of the participants was 33 years with a range of 4 to 85 years. More than half (65.6%) of the patients were married and 244 (61.8%) of them never attended formal education. Farmers accounted relatively the highest percentage (27.9%) followed by housewives (26.6%) and students (14.4%). More than half (66.1%) of the respondents were protestant religion followers and rural residents (64.1%). The mean monthly income of the study participants was 449 Ethiopian birr (21.4 USD) with a minimum of having no income to a maximum of 3600 Ethiopian birr (171 USD) (Table 1).

**Table 1:** Bivariate analysis of socioeconomic and behavioral factors with patient delay in TB diagnosis

Variable	Value	Number (%)	Delay ≤ 21 days n (%)	Delay > 21 days n (%)	Crude OR (CI)	P value
Age	< 19	32 (8.1)	9 (28.1)	23 (71.9)	2.31 (1.06–4.97)	0.49
	19–35	226 (57.2)	96 (42.5)	130 (57.5)	1.22 (0.94–1.59)	
	35–45^a^	76 (19.2)	36 (47.4)	40 (52.6)	1.00	
	> 45	61 (15.4)	24 (39.3)	37 (60.7)	1.39 (0.83–2.32)	
Sex	Male^a^	224 (56.7)	92 (41.1)	132 (58.9)	1.00	0.75
	Female	171 (43.3)	73 (42.7)	98 (57.3)	1.07 (0.82–1.40).l	
Education	Uneducated	128 (32.4)	69 (53.9)	59 (46.1)	0.98 (0.69–1.38)	0.88
	Can read and write	116 (29.4)	76 (65.5)	40 (34.5)	1.58 (1.08–2.32)	
	Primary school	118 (29.9)	67 (56.8%)	41 (43.2)	1.09 (0.76–1.58)	
	Secondary and above^a^	33 (8.3)	18 (54.5)	15 (45.5)	1.00	
Occupation	Unemployed	30 (7.6)	13 (43.3)	17 (56.7)	10.08 (4.85–20.69)	0.001*
	Government employee^a^	36 (9.1)	32 (88.9)	4 (11.1%)	1.00	
	Farmer	110 (27.8)	40 (36.4)	70 (63.6)	13.46 (9.15–19.85)	
	Private worker	51 (12.9)	26 (51.0)	25 (49.0)	7.38 (4.31–12.77)	
	Student	57 (14.4)	18 (31.6)	39 (68.4)	16.69 (9.54–29.15)	
	House wife	105 (26.6)	33 (31.4)	72 (68.6)	16.77 (11.15–25.31)	
	Daily laborer	6 (1.5)	3 (50.0)	3 (50.0)	7.69 (1.54–38.08)	
Religion	Orthodox	50 (12.6)	30 (60.0)	20 (40.0)	1.56 (0.88–2.72)	0.09*
	Catholic	27 (6.9)	10 (37.0)	17 (63.0)	3.95 (1.81–8.63)	
	Protestant	261 (66.1)	85 (32.6)	176 (67.4)	4.81 (3.26–6.23)	
	Muslim^a^	57 (14.4)	40 (70.2)	17 (29.8)	1.00	
Residence	Urban	142 (36.0)	41 (28.9)	101 (71.1)	2.36 (1.64–3.40)	0.0001*
	Rural^a^	253 (64.0)	124 (49.0)	129 (51.0)	1.00	
Monthly income	< 100 EB	78 (19.7)	19 (24.4)	59 (75.6)	3.38 (2.01–5.66)	0.0001*
	100–500 EB	223 (56.5)	97 (43.5)	126 (56.5)	1.41 (1.08–1.84)	
	> 500 EB^a^	94 (23.8)	49 (52.1)	45 (47.9)	1.00	
Distance from HF	≤ 5 km^a^	300 (75.9)	133 (44.3)	167 (55.7)	1.00	0.07*
	> 5 km	95 (24.1)	32 (43.7)	63 (66.3)	1.57 (1.03–2.41)	
Knowledge about TB transmission	Good^a^	356 (90.1)	142 (39.9)	214 (60.1)	1.00	0.02*
	Poor	39 (9.9)	23 (59.0)	16 (41.0)	0.46 (0.25–0.88)	
Knowledge about TB symptoms	Good^a^	317 (80.2)	132 (41.6)	185 (58.4)	1.00	0.91
	Poor	78 (19.8)	33 (42.3)	45 (57.7)	1.04 (0.71–1.54)	
Belief in the curability of TB	No	25 (6.3)	18 (72.0)	7 (28.0)	0.26 (0.11–0.61)	0.003*
	Yes^a^	370 (93.7)	147 (39.7)	223 (60.3)	1.00	
Comfort with DOTS service	No	105 (26.6)	36 (34.3)	69 (65.7)	1.54 (1.02–2.30)	0.06*
	Yes^a^	290 (73.4)	129 (44.5)	161 (55.5)	1.00	

* Statistically significant when P < 0.2

^a^Reference category

#### Relationship between patient delay in TB diagnosis and contributing factors

The median time interval between the day a patient first experienced one of the pulmonary symptoms and sought medical advice was 30 days with the earliest and most delayed span of 5 and 120 days, respectively. In other words, more than half (58.2%) of the participants got treatment after 21 days of delay since they first experienced tuberculosis symptoms. The difference in delay between males and females was comparable in that 132 (58.9%) of the male patients and 98 (57.3%) of the female patients delayed for more than 21 days to be diagnosed. Youngsters (less than 19 years of age) (71%) and elderly patients aged more than 45 years (60%) took relatively longer time to go to health facility in seeking treatment for their symptoms though the difference was not statistically significant (P = 0.49).

However, there was statistically significant difference in time elapse before getting treatment in terms of residence in that urban residents were 2.36 times more likely to delay to seek treatment than rural residents (P = 0.03, OR 2.36, CI 1.64–3.40). Similarly, religion was also found to be an independent predictor of tuberculosis diagnosis delay. 67.4% of the protestant and 63% of the catholic religion followers stayed undiagnosed for more than 21 days since they first observed TB symptoms [P = 0.0001, OR 1.24, CI 1.73–7.00]. Likewise, participants having a monthly income of less than 100 Ethiopian Birr (< 5 USD) were 3.38 times more likely to delay to get TB treatment than those earning more [P = 0.0001, OR 3.38, CI 2.01–5.66]. Patients who lack comfort with DOTS service also delayed more (65.7%) than those patients who were comfortable with the service (55.5%) [P = 0.003, OR 1.54, CI 1.02–2.30] (Table 2).

**Table 2:** Multivariate analysis of the relationship between patient delay to TB case detection and contributing factors

Variable	Value	Number (%)	Time ≤ 21 days n (%)	Delay > 21 days n (%)	Adjusted OR (CI)	P value
Residence	Urban	142 (36.0)	41 (28.9)	101 (71.1)	2.36 (1.64–3.40)	0.03*
	Rural^a^	253 (64.0)	124 (49.0)	129 (51.0)	1.00	
Occupation	Unemployed	30 (7.6%)	13 (43.3%)	17 (56.7%)	10.08 (4.85–20.69)	0.08
	Government employee^a^	36 (9.1%)	32 (88.9%)	4 (11.1%)	1.00	
	Farmer	110 (27.8%)	40 (36.4%)	70 (63.6%)	13.46 (9.15–19.85)	
	Private worker	51 (12.9%)	26 (51.0%)	25 (49.0%)	7.38 (4.31–12.77)	
	Student	57 (14.4%)	18 (31.6%)	39 (68.4%)	16.69 (9.54–29.15)	
	House wife	105 (26.6%)	33 (31.4%)	72 (68.6%)	16.77 (11.15–25.31)	
	Daily laborer	6 (1.5%)	3 (50.0%)	3 (50.0%)	7.69 (1.54–38.08)	
Religion	Orthodox	50 (12.6)	30 (60.0)	20 (40.0)	1.43 (0.81–2.50)	0.0001*
	Catholic	27 (6.9)	10 (37.0)	17 (63.0)	2.67 (0.92–7.73)	
	Protestant	261 (66.1)	85 (32.6)	176 (67.4)	1.24 (1.73–7.00)	
	Muslim^a^	57 (14.4)	40 (70.2)	17 (29.8)	1.00	
Monthly income	< 100 EB	78 (19.7)	19 (24.4)	59 (75.6)	3.38 (2.01–5.66)	0.0001*
	100–500 EB	223 (56.5)	97 (43.5)	126 (56.5)	1.92 (1.00–3.73)	
	> 500 EB^a^	94 (23.8)	49 (52.1)	45 (47.9)	1.00	
Distance from HF	≤ 5 km^a^	300 (75.9)	133 (44.3)	167 (55.7)	1.00	0.05
	> 5 km	95 (24.1)	32 (43.7)	63 (66.3)	1.57 (1.03–2.41)	
Knowledge about TB transmission	Good^a^	356 (90.1)	142 (39.9)	214 (60.1)	1.00	0.25
	Poor	39 (9.9)	23 (59.0)	16 (41.0)	0.44 (0.23–0.84)	
	Yes^a^	370 (93.7)	147 (39.7)	223 (60.3)	1.00	
Ever screened for HIV	No	85 (21.5)	27 (31.8)	58 (68.2)	1.72 (1.09–2.71)	0.08
	Yes^a^	310 (78.5)	138 (44.5)	172 (55.5)	1.00	
Belief in the curability of TB	No	25 (6.3)	18 (72.0)	7 (28.0)	0.06 (0.02–0.14)	0.0001*
	Yes^a^	370 (93.7)	147 (39.7)	223 (60.3)	1.00	
Comfort with DOTS	No	105 (26.6)	36 (34.3)	69 (65.7)	1.54 (1.02–2.30)	0.003*
	Yes^a^	290 (73.4)	129 (44.5)	161 (55.5)	1.00	

* Statistically significant when P < 0.05

^a^Reference category

### Discussion

This study documented high patient delay in seeking tuberculosis diagnosis in Hadiya Zone with a median delay of 30 days. More than half (58.2%) of the patients got diagnosed after spending more than WHO recommended time i.e. 21 days since they first experienced at least one tuberculosis symptoms [7]. This is comparable with the finding of a study in Brazil which reported the median patient delay of 30 days [8] but lower than that of Nigerian study where 83% of patients presented in health facilities after a month or more from the onset of their symptoms [9].

On the other hand, lower percentage of delay was indicated in Tanzania i.e. 38.4% of patients delayed to seek TB health care in fact the study considered 13 days as a cut off point for patient delay which is 8 days shorter than the present study [10]. A study conducted in 2002 in Ethiopia reported the median patient delay of 60 which is twice of the present finding. Whereas subsequent studies conducted in 2007 and 2014 in Western and Northern part of the country reported concordant results with the current study i.e. 28 and 30 days of delay, respectively [11, 12].

In Ethiopia and elsewhere, patient delay has been associated with various socioeconomic, patient awareness and health service related factors [9–14]. The present study also revealed different socioeconomic and attitudinal factors such as residence, religion, monthly income, misconception about TB curability and perception towards DOTS service as independent predictors of patient delay in TB diagnosis. In contrary to most studies conducted which reported rural residence as a risk factor for TB diagnosis delay [15–18], urban residents delayed more than their rural counterparts in this study. The studies mentioned low access to health service and information in rural areas as a reason for the delay. Conversely, majority of the patients in the current study were fairly knowledgeable about the disease and remoteness of their residence was not found to be associated with diagnosis delay. Thus, longer delay in TB diagnosis among urban population in this study might be due to misconception attributed to strong belief in curability of the disease so that it could be healed whenever they get treatment. The low diagnosis delay time among rural residents could also be as result of regular and intensive health education offered by health extension workers about the disease which is not yet actively implemented in urban settings in the country.

Similarly, more than half of the patients perceived that symptoms would disappear without treatment while more than quarter of them got treatment for their symptoms by themselves and in traditional ways. This was further strengthened by multivariate analysis that religion was found to be one of the independent determinants of TB diagnosis delay. Mesfin et al. [19] also reported similar findings from Northern Ethiopia where treatment by spiritual and private practitioners contributed to 79% of the delay [19]. A study in Zimbabwe as well revealed that TB patients neglected the symptoms and those taking self-medication were the most common reasons for patient delay [20].

It has continually been noticed that level of income is an important determinant of patient’s early tuberculosis diagnosis [21, 22]. Comparable result has been documented in this study too where participants earning less were more likely to delay to get TB treatment than those earning more. This might mainly be due to the fact that people with better income hesitate less to spend money for transportation and other expenditure associated with it. Some people might not have sufficient information that TB diagnosis and treatment is freely available either in private or public health facilities and hence worry of getting money for that. Moreover, some of them might not even want to waste their crucial time that they need it to earn income for their family’s survival [23]. This can also be linked to dissatisfaction with the widespread introduction of DOTS service which requires patient’s daily visit so that it is perceived to be time consuming and a risk of considerable loss of income [24].

### Conclusions

The current study identified longer delay for TB patients to get diagnosed and then treated relative to WHO recommended time [7]. The aggravating factors found to be misperception on curability of the disease, religious views, low income and discomfort with DOTS service. Thus, there is a need for more robust information dissemination strategy to ultimately change people’s views that tuberculosis can only be cured when diagnosed and treated promptly. Community should also be aware of the opportunity that tuberculosis diagnosis and treatment is free of charge in any private and public health facilities. Furthermore, decentralization of DOTS service to peripheral health facilities such as health posts should be strengthened. Urban health extension work should also be further strengthened as that of more progressive accomplishments observed in rural health extension.

## Limitations

The study is not free of recall bias as it completely relied on patients’ report. Thus, there could be over or under reporting of delay time. In addition, patients on treatment may have some unique features that people in the community do not have. Consequently, there could be misrepresentation of the actual situation in the community to some extent. Moreover, patient delay documented in this study was not triangulated with possible health service delay which could have its own determinants.

## Abbreviations

CI: confidence interval
DOTS: directly observed treatment short course
E.C: Ethiopian Calendar
HIV: Human Immunodeficiency Virus
MDR: multi drug resistant
MPH: Master of Public Health
OR: odds ratio
RERE: Research Ethical Review Committee
SNNPR: Southern, Nations, Nationalities and Peoples Region
TB: tuberculosis
WHO: World Health Organization
